# Integrative analysis identifies cancer cell-intrinsic RARRES1 as a predictor of prognosis and immune response in triple-negative breast cancer

**DOI:** 10.3389/fgene.2024.1360507

**Published:** 2024-03-12

**Authors:** Zhengheng Yu, Hongjin Liu, Jingming Ye, Yinhua Liu, Ling Xin, Qian Liu, Yuanjia Cheng, Lu Yin, Ling Xu

**Affiliations:** ^1^ Department of Thyroid and Breast Surgery, Peking University First Hospital, Beijing, China; ^2^ Department of Urology, National Cancer Center/National Clinical Research Center for Cancer/Cancer Hospital, Chinese Academy of Medical Sciences and Peking Union Medical College, Beijing, China

**Keywords:** triple-negative breast cancer, tumor microenvironment, single-cell RNA sequencing, *RARRES1*, immune response, bioinformatic analysis

## Abstract

Triple-negative breast cancer (TNBC) is a subtype of breast cancer with poor prognosis and limited treatment options. Although immune checkpoint inhibitors (ICIs) have been proven to improve outcomes in TNBC patients, the potential mechanisms and markers that determine the therapeutic response to ICIs remains uncertain. Revealing the relationship and interaction between cancer cells and tumor microenvironment (TME) could be helpful in predicting treatment efficacy and developing novel therapeutic agents. By analyzing single-cell RNA sequencing dataset, we comprehensively profiled cell types and subpopulations as well as identified their signatures in the TME of TNBC. We also proposed a method for quantitatively assessment of the TME immune profile and provided a framework for identifying cancer cell-intrinsic features associated with TME through integrated analysis. Using integrative analyses, *RARRES1* was identified as a TME-associated gene, whose expression was positively correlated with prognosis and response to ICIs in TNBC. In conclusion, this study characterized the heterogeneity of cellular components in TME of TNBC patients, and brought new insights into the relationship between cancer cells and TME. In addition, *RARRES1* was identified as a potential predictor of prognosis and response to ICIs in TNBC.

## 1 Introduction

Worldwide, female breast cancer is the most common malignancy and accounts for the highest mortality rate in woman among cancers (Sung et al., 2021). Triple-negative breast cancer (TNBC), which is a subtype of breast cancer with poor prognosis and limited treatment options, accounts for about 15%–20% of all breast cancers (Cancer Genome Atlas Network, 2012). Characterized by the lack of estrogen receptor (ER), progesterone receptor (PR), and human epidermal growth factor receptor 2 (HER2) expression, TNBC has limited therapeutic options (Bianchini et al., 2022).

Whether in localized or advanced stage, chemotherapy is currently the main first-line treatment option for TNBC. However, the prognosis for triple-negative breast cancer is the worst among all major subtypes of breast cancer. Immune checkpoint inhibitors (ICIs) have made important breakthroughs in the treatment of a variety of solid tumors, including TNBC (Bianchini et al., 2022). However, only a subset of TNBC patients can benefit from treatment with ICIs. Therefore, it is a major issue to identify the potential mechanisms and markers that determine the therapeutic response to ICIs.

The tumor microenvironment (TME) is a complex ecosystem that includes multiple cell types such as cancer cells, immune cells, stromal cells, vascular cells, surrounded by the extracellular matrix (de Visser and Joyce, 2023). Various types of cells in the TME have important roles in tumorigenesis, progression, and response to treatment, which can be either tumor-suppressive or tumor-promotive. It has been demonstrated that the function of cells in TME can be regulated by the intrinsic features of cancer cells (Wellenstein and de Visser, 2018). Thus, revealing the relationship and interaction between cancer cells and TME could be helpful in predicting treatment efficacy and developing novel therapeutic agents.

**FIGURE 1:**
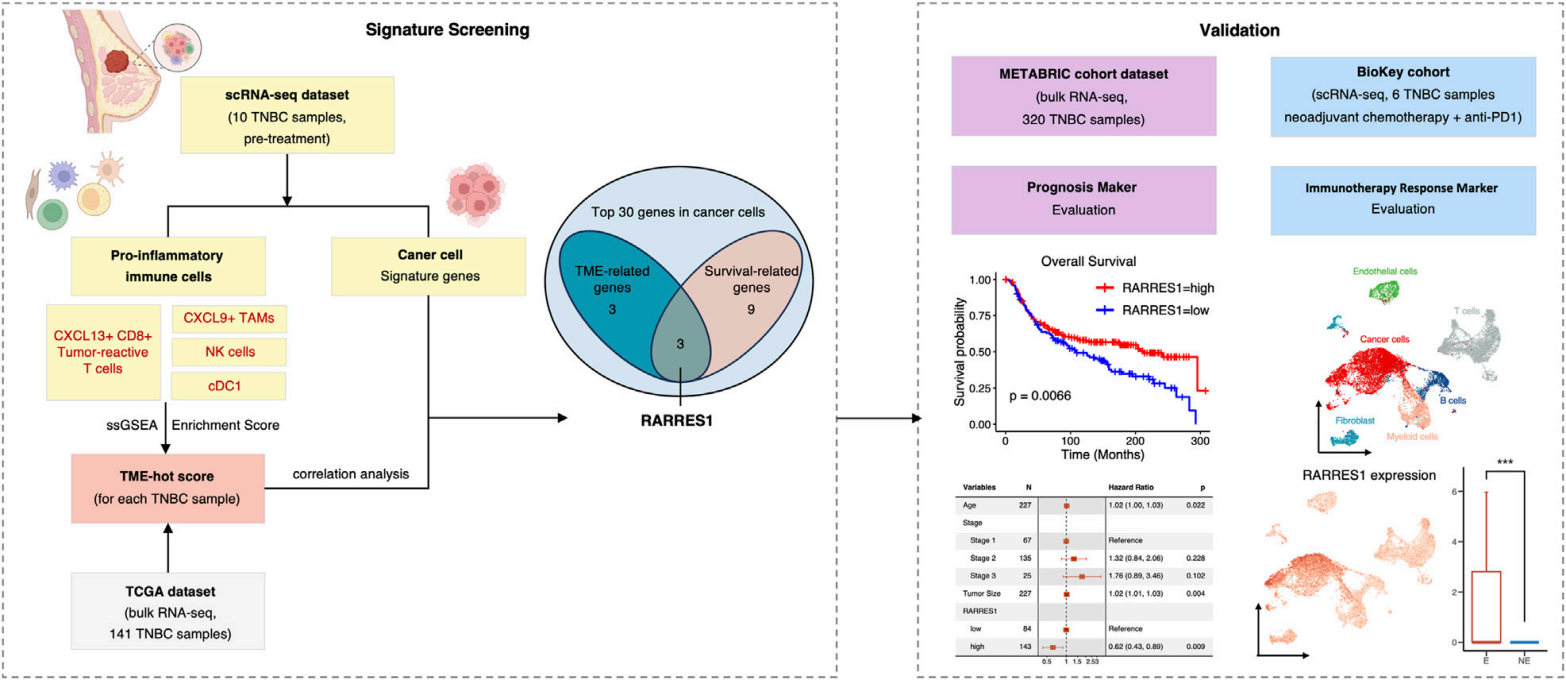
The flowchart of the present study.

Recent developments in single-cell RNA sequencing (scRNA-seq) have made it possible to analyze the heterogeneity, functional status, interactions, and evolving trajectories of cells in the TME at single-cell resolution (Bassez et al., 2021; Huang and Zhang, 2021b; Wu et al., 2021; Yan et al., 2021; Zhao et al., 2022). Several studies have revealed the heterogeneity of immune cells, especially T cells and Myeloid cells, in TNBC, based on single-cell sequencing. For example, CXCL13+ tumor reactive T cells were the only subpopulation that associated with favorable response to ICIs (Zhang et al., 2021). In addition, high level of CXCL9+ tumor-associated macrophages (TAMs) was associated with favorable prognosis, implying that this subpopulation of macrophage may activate anti-tumor immune responses (Bill et al., 2023). In contrast, SPP1+ or TREM2+ TAMs play an important role in the immune escape of the tumor cells (Nalio Ramos et al., 2022; Bill et al., 2023). Herein, we comprehensively profiled cell types and subpopulations as well as identified their signatures in the TME of TNBC based on scRNA-seq data. We also proposed a method for quantitatively assessment of the TME immune profile and provided a framework for identifying cancer cell-intrinsic features associated with TME through integrated analysis. In addition, we characterized *RARRES1* as a predictor of prognosis and response to ICIs in TNBC (Figure 1). These results bring new insights into the relationship between cancer cells and TME and are expected to provide groundwork for the development of biomarkers and new therapies for TNBC.

## 2 Materials and methods

### 2.1 Data source

Four independent published breast cancer cohorts were included in this study. Cohort 1 was obtained from the GSE176078 scRNA-seq dataset from the Gene Expression Omnibus (GEO) database (http://www.ncbi.nlm.nih.gov/geo/), containing 11 ER+, 5HER2+ and 10 TNBC cases (Wu et al., 2021). Cohort 2 consisted of 141 patients with primary TNBC in the Cancer Genome Atlas (TCGA) database who were profiled using bulk RNA-seq (Hutter and Zenklusen, 2018). Cohort 3 is the Molecular Taxonomy of Breast Cancer International Consortium (METABRIC) cohort of 1980 breast cancer patients, including 320 triple-negative breast cancer patients, with mRNA Expression matrix profiled using Illumina HT-12 v3 microarray (Curtis et al., 2012). The TCGA and METABRIC datasets were downloaded from cBioPortal database (https://www.cbioportal.org/) (Cerami et al., 2012). Cohort 4 was a scRNA-seq dataset from the BioKey study (http://biokey.lambrechtslab.org), in which 3 ER+, 1 HER2+, and 6 TNBC patients received neoadjuvant chemotherapy followed by pembrolizumab before surgery (Bassez et al., 2021). In all cohorts described above, data of TNBC patients were divided for analysis.

### 2.2 Quality control, integration, dimensionality reduction, and clustering of scRNA-seq data

The Seurat package (version 4.4.0) in R software (https://www.r-project.org/, version 4.2.3) was used for analyses of scRNA-seq data (Hao et al., 2021). After import into the R software, scRNA-seq data was split by sample. Cells with less than 200 genes, more than 7000 genes, or more than 15% mitochondrial gene expression were removed. Normalized data of the remaining cells was integrated with “FindIntegrationAnchors” and “IntegrateData” function using default parameters. Subsequently, linear dimensional reduction was performed on scaled data using principal component analysis (PCA). Cells were clustered with the first 35 principal components and a resolution of 0.8. For re-clustering immune cells, the first 20 and 15 principal components were used for T & NK cells, and myeloid cells, respectively, with a resolution of 0.9. The uniform manifold approximation and projection (UMAP) method was used to visualize all data sets.

### 2.3 Annotation of cell clusters and identification of signature genes

We used the “FindAllMarkers” function to select differentially expressed genes (DEGs). The markers of cell types and clusters for annotation were collected from multiple published data and were cross-referenced with DEGs of each cluster. To determine the signature genes for each cell type and cluster, we first selected the top 15 DEGs and then removed some genes from them that were repeated in multiple clusters according to the following principles. Top 10 DEGs of T cells and myeloid cells were defined as general T genes and general myeloid genes. For clusters in T cells, general T genes that appeared in both Treg and other cell clusters were removed. For tumor-associated macrophages (TAMs) clusters, general myeloid genes that occurred in more than two clusters were excluded. For three cDC clusters, genes that appeared in all 3 clusters were deleted.

### 2.4 TME score analysis

Enrichment score (ES) of each cell type or cluster for each sample was calculated with the single-sample gene set enrichment analysis (ssGSEA) method implemented in the GSVA (version 1.44.5) package based on signature genes selected above (Barbie et al., 2009). We then developed a TAM score, a T score and a TME-hot score based on the ES. The TAM score measures the ratio of TAM_CXCL9 score and TAM_SPP1/TREM2 score. The T score calculates the sum of CD8_Ttr_CXCL13 score, CD8_Teff_GZMB score, and Tact_IFI6 score. While the TME-hot is defined as the sum of the TAM score, the T score, the ES of NK cells, and the ES of cDC1 cluster. The subpopulations and signatures used to calculate the TME-hot score was shown in Supplementary Table S3.

### 2.5 Statistical analysis

R software (version 4.4.0) was employed for all of the statistical analyses and visualization. The prognostic value of variables was analyzed by using Kaplan-Meier survival curve and Cox regression analysis in TNBC cohorts. The log-rank test was used to determine the significance of different survival curves. Linear correlations between continuous variables were analyzed with Pearson correlation coefficient.

## 3 Results

### 3.1 Cellular composition of TME in TNBC

**FIGURE 2:**
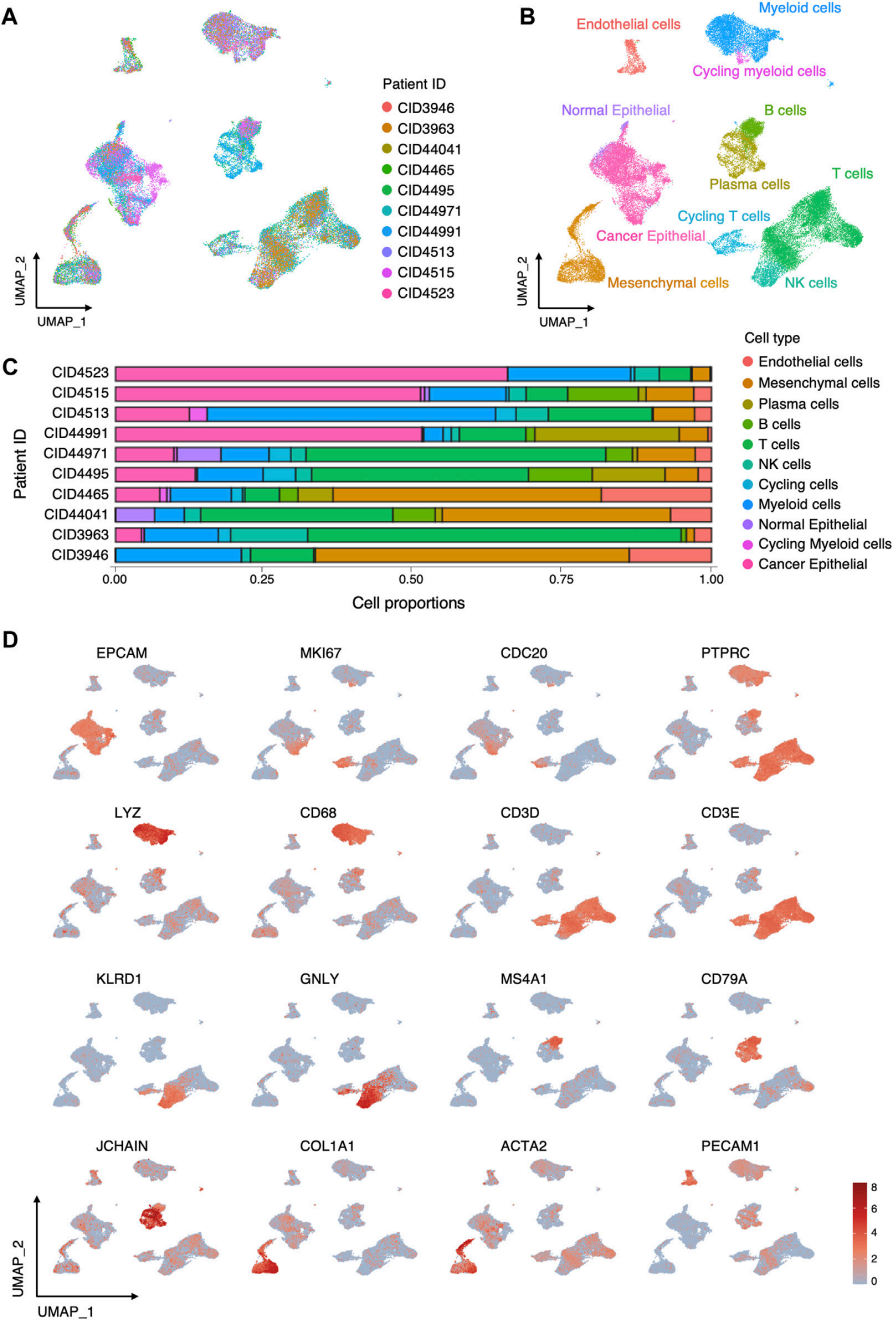
Cellular composition of TME in TNBC. **(A)** UMAP visualization of 40834 cells integrated across 10 TNBC. **(B)** UMAP visualization of major cell types in TNBC. **(C)** Relative proportions of cell types in each sample. **(D)** Log-normalized expressions of selected marker genes in major cell types.

To elucidate the cellular composition of the TME in TNBC, we acquired and reanalyzed a scRNA-seq dataset with 10 TNBC samples. After quality control, data from 40834 high-quality cells were retained for subsequent analyses. Data from individual samples were integrated and clustered with the canonical correlation analysis and UMAP algorithm (Figure 2A). According to canonical lineage marker, 9 major cell types were annotated (Figures 2B, C), including epithelial cells (*EPCAM*), T cells (*PTPRC, CD3D, CD3E*), natural killer (NK) cells (*NKG7, GNLY, KLRD1*), B cells (*MS4A1, CD79A*), plasma cells (*JCHAIN*,*CD79A*), myeloid cells (*LYZ, CD68*), mesenchymal cell (*COL1A1, ACTA2*), endothelial cells (*PECAM1*), and cycling cells (*MKI67, CDC20, TOP2A*). Subsequently, cancer cells were distinguished from normal epithelial cells according to the copy number variant estimated by inferCNV (Figure 2B, Supplementary Figure S1). Proportion of cell types in each sample was shown in Figure 2D.

**FIGURE 3:**
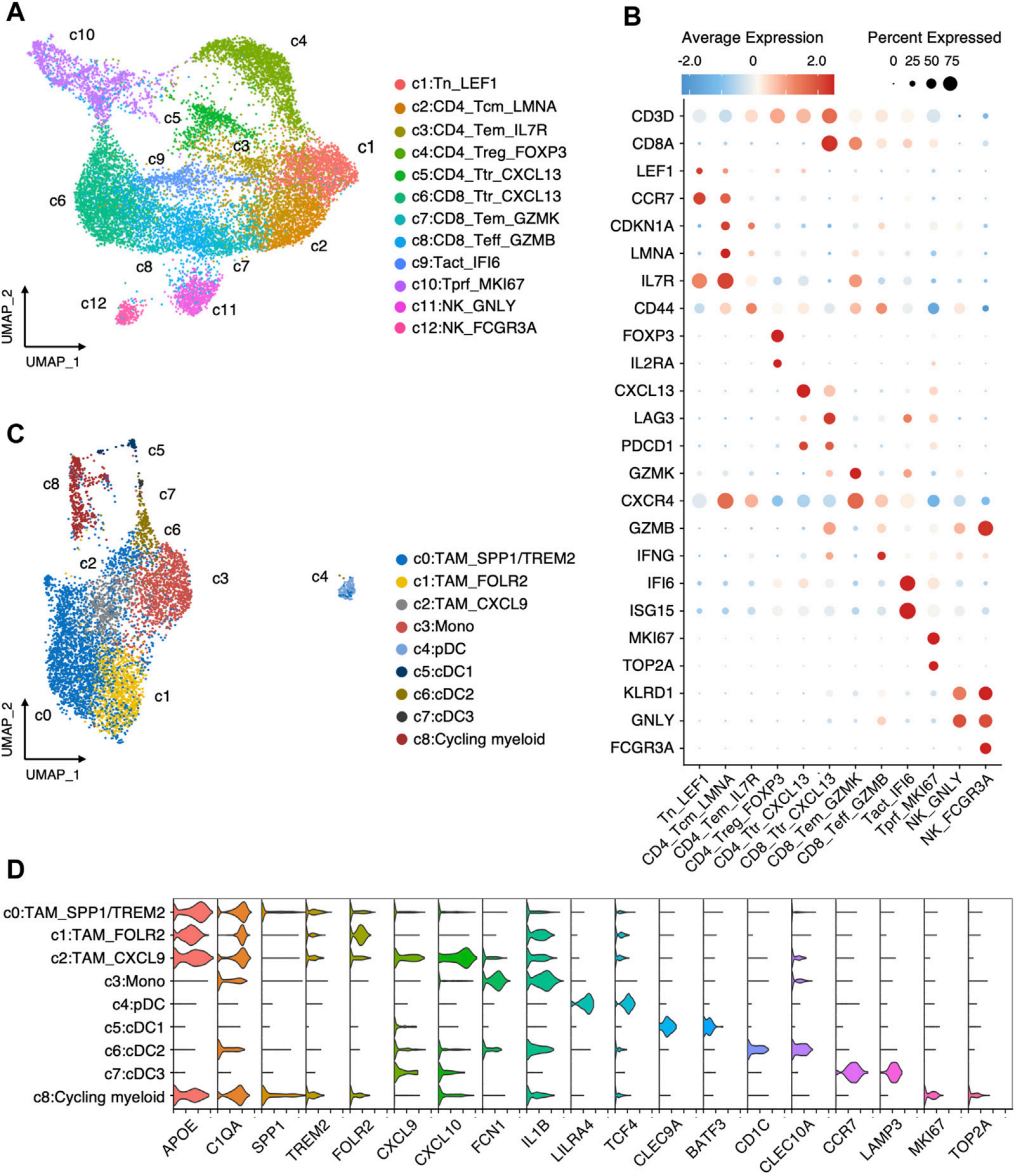
Landscape of immune cells in TNBC. **(A)** Re-clustering T/NK cells into 12 clusters. **(B)** Average expression of canonical markers across T/NK cell clusters. **(C)** Re-clustering myeloid cells into 9 clusters. **(D)** Log-normalized expressions of selected marker genes in myeloid cell clusters.

We further inspected the cellular heterogeneity in the immune microenvironment of TNBC through re-clustering T cells, NK cells, and Myeloid cells. In T cells and NK cells, 12 clusters were identified (Figures 3A, B), including two tumor-reactive T (Ttr) cell cluster (c5:CD4_Ttr_CXCL13 and c6:CD8_Ttr_CXCL13) with high expression of *CXCL13* and *PDCD1*; one regulatory T (Treg) cell cluster (c4:CD4_Trg_FOXP3) marked by *FOXP3* and *IL2RA*; three clusters (c1:Tn_LEF1, c9:Tact_IFI6, and c10:Tprf_MKI67) with both CD4^+^ and CD8^+^ T cells comprising naive (Tn), activated (Tact), and proliferative (Tprf) T cells; four classical CD4^+^ and CD8^+^ T cell clusters (c2:CD4_Tcm_LMNA, c3:CD4_Tem_IL7R, c7:CD8_Tem_GZMK, and c8:CD8_Teff_GZMB) comprising central memory (Tcm), effector (Teff), and effector memory (Tem) T cells; and two NK cell clusters (c11:NK_GNLY, c12:NK_FCGR3A). In myeloid cells (Figures 3C, D), we identified three tumor-associated macrophages (TAMs) clusters (c0:TAM_SPP1/TREM2, c1:TAM_FOLR2, c2:TAM_CXCL9) according to the expression of *SPP1*, *TREM2*, *FOLR2*, and *CXCL9*; monocyte (c3:Mono) marked by FCN1 and IL1B expression; plasmacytoid dendritic cells (pDC) with high expression of LILR44 and TCF4; three conventional dendritic cells (cDCs) characterized by high expression of either CLEC9A (c5:cDC1), CD1C (c6:cDC2), or LAMP3 (c7:cDC3); and a MKI67 high cycling cluster (c9:Cycling myeloid).

### 3.2 Identification of TME-associated cancer cell-intrinsic genes

To quantitatively evaluate the TME immune profile of individual TNBC samples, we obtained signature gene sets (Supplementary Table S1) of each cell type or cell cluster by differential gene analysis, which can be used for ssGSEA. Firstly, we analyzed the top 15 DEGs as signature genes for each cell type and cluster. Then, genes co-existing in multiple cell clusters were removed (Supplementary Table S2) according to the following criteria. For signature genes of clusters in T cells, general T genes that appeared in both Treg and other cell clusters were removed. For signature genes of TAM clusters, general myeloid genes that occurred in more than two clusters were excluded. For signature genes of three cDC clusters, genes that appeared in all 3 clusters were deleted.

**FIGURE 4:**
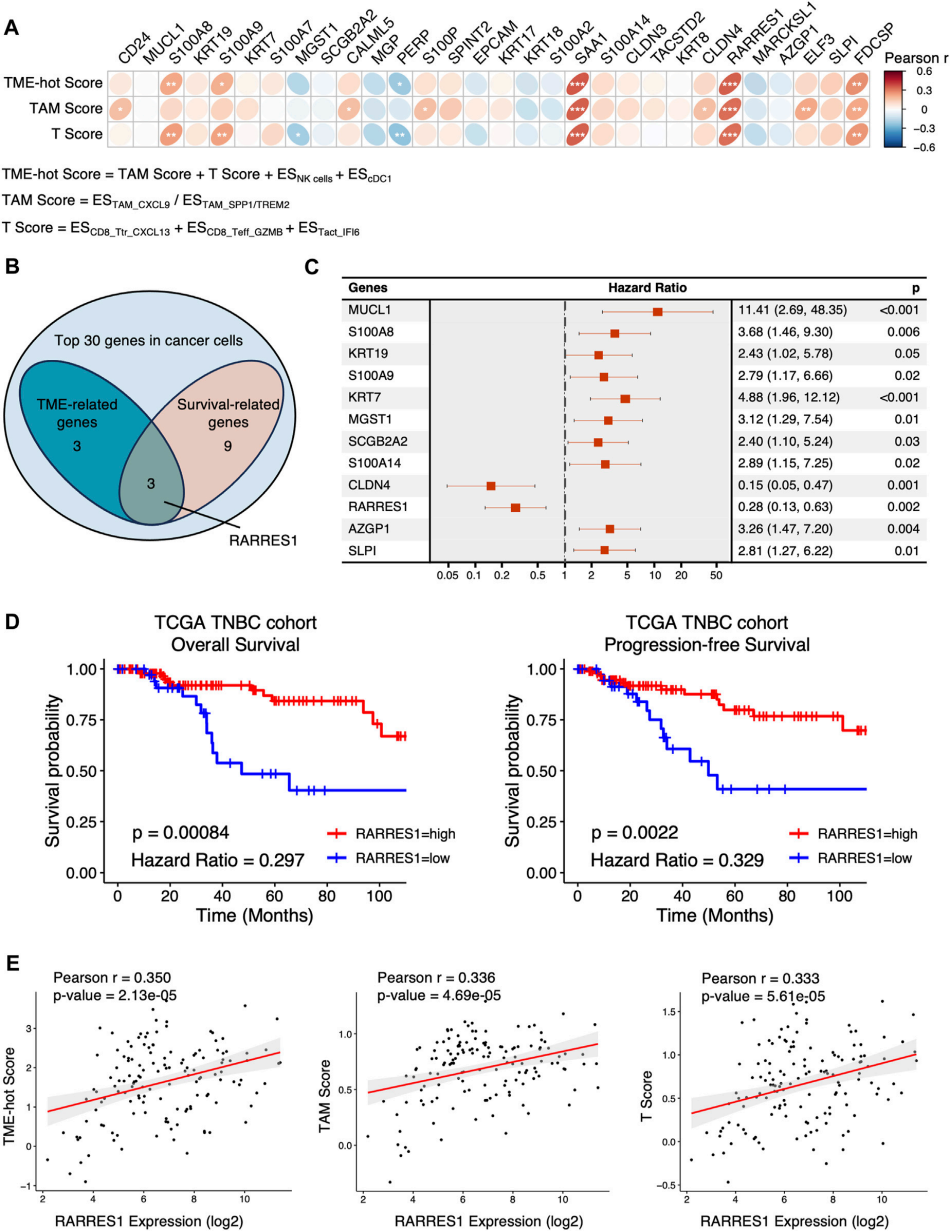
Identification of TME-associated cancer cell-intrinsic genes. **(A)** The Pearson correlation between the top 30 genes in cancer cells and TME-hot score. ES = enrichment score, * = 0.01 < *p* < 0.05, ** = 0.001 < *p* < 0.01, *** = *p* < 0.001. **(B)** Three intersecting genes between TME-related genes and survival-related genes in Venn diagram. **(C)** Forest plot showing the 12 cancer cell-intrinsic genes significantly associated with overall survival using univariate Cox regression in TCGA cohort. **(D)** Overall survival and progression-free survival of TNBC patients with low or high expression of RARRES1 in TCGA cohort. **(E)** The correlation between RARRES1 expression and TME-hot score, TAM score, and T score, respectively. ES = enrichment score.

Subsequently, ES of each cell type and cluster for each TNBC sample in TCGA cohort was calculated using ssGSEA method. We assessed the TME-hot score, T score, and TAM score for each TNBC sample (Figure 4A; Supplementary Table S3). By investigating the correlation between the TME-hot score and the top 30 DEGs of cancer cells, six TME-associated genes were picked out (Figures 4A, B). Three genes were then identified as being associated with survival by using univariate Cox regression analyses (Figures 4B, C), including *RARRES1*, *S100A8*, and *S100A9*.

We then focused on the role of *RARRES1* in TNBC, because S100A8 and S100A9 were positively correlated with TME-hot score but negatively correlated with overall survival, which was contradictory. High *RARRES1* expression is associated with not only better overall survival but also better progression-free survival in TCGA TNBC patients (Figure 4D). Furthermore, correlation analysis demonstrated that high *RARRES1* expression was associated with not only better TME-hot score, but also better TAM score and T score (Figures 4A, E). In summary, high *RARRES1* expression was associated with “hot” TME and good prognosis in TCGA cohort.

### 3.3 *RARRES1* is a predictor of prognosis and immune response in TNBC

**FIGURE 5:**
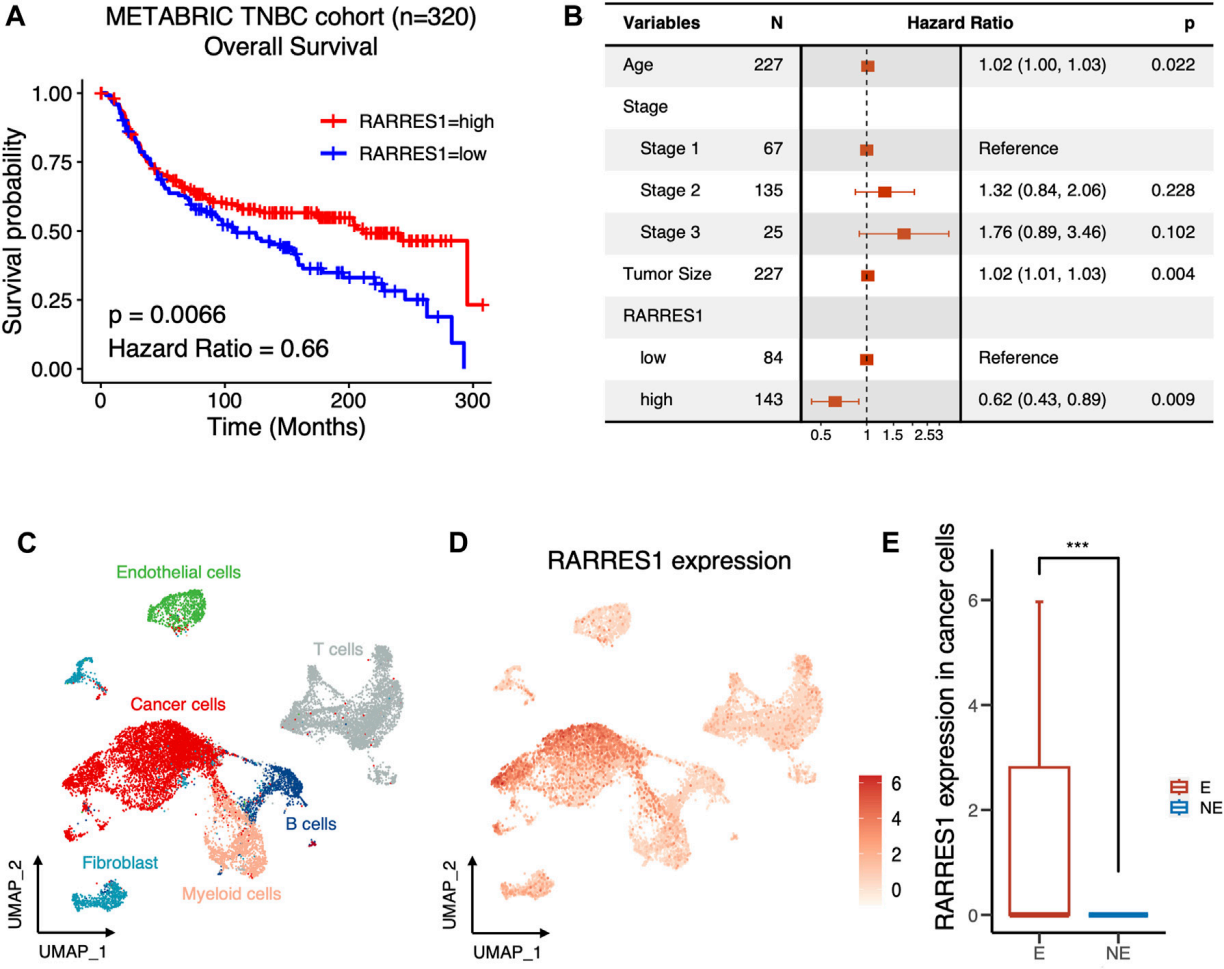
*RARRES1* is a predictor of prognosis and immune response in TNBC. **(A)** Kaplan–Meier curves showing the overall survival of TNBC patients with low or high expression of *RARRES1* in METABRIC cohort. **(B)** Multivariate Cox regression of *RARRES1* expression and other clinical prognostic parameters in METABRIC cohort. **(C)** UMAP visualization of cells from 6 pre-treatment TNBC patients in BioKey study. **(D)** Log-normalized expressions of RARRES1 in major cell types. **(E)** *RARRES1* expression in cancer cells between patients with **(E)** or without (NE) T cell clonotype expansion. *** = *p* < 0.001.

The predictive role of *RARRES1* for clinical outcomes in TNBC was further validated in the METABRIC cohort with 320 TNBC patients. Kaplan–Meier survival analysis suggested that patients with high *RARRES1* expression had significantly better overall survival (*p* = 0.0066) than those with low *RARRES1* expression (Figure 5A). Multivariate Cox regression revealed that the *RARRES1* expression was an independent prognostic indicator, with a hazard ratio (HR) of 0.66 and 0.62 in the univariate and multifactorial analyses, respectively (*p* < 0.001) (Figure 5B). In addition, RARRES1 expression was not related to tumor stage of TNBC (Supplementary Figure S2).

ICIs have made important breakthroughs in the treatment of a variety of solid tumors, including TNBC. However, only a subset of TNBC patients can benefit from treatment with ICIs. Therefore, it is a major issue to identify the potential mechanisms and markers that determine the therapeutic response to ICIs. Therefore, to clarify whether *RARRES1* is associated with response to immunotherapy, we investigated pretreatment *RARRES1* levels in the scRNA-seq data set of the BioKey cohort (NCT03197389), in which patients with TNBC received neoadjuvant chemotherapy followed by pembrolizumab before surgery. As expected, *RSRRES1* was highly expressed only in cancer cells (Figures 5C, D) and significantly enriched in patients with T cells clonotype expansion (Figure 5E), which represents a better immune response. Overall, the above results indicated *RARRES1* as a potential predictor of prognosis and treatment response to ICIs in TNBC.

### 3.4 *RARRES1* is a TNBC-specific predictor of prognosis and immune response

**FIGURE 6:**
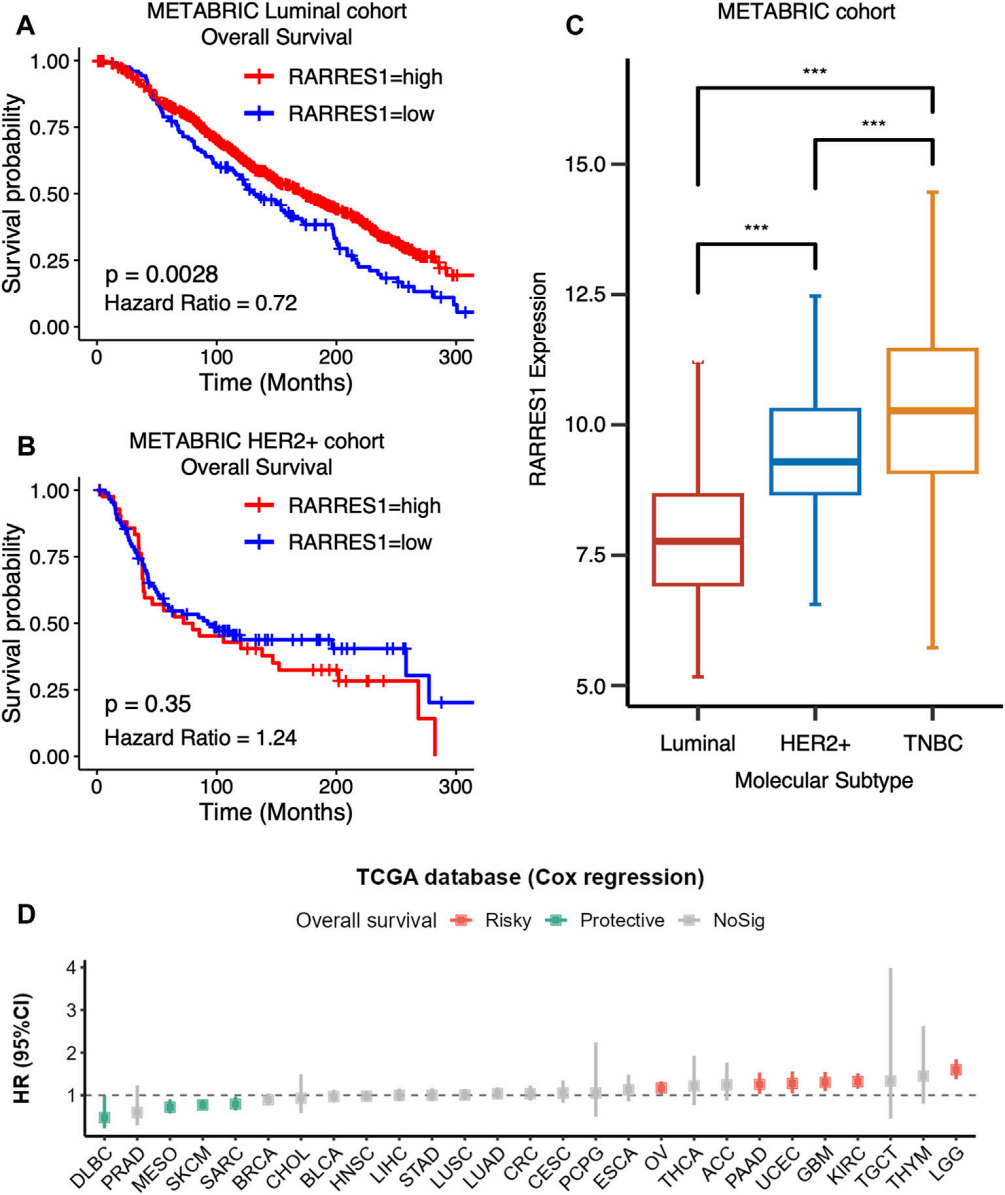
RARRES1 is a TNBC-specific predictor of prognosis. **(A)** Kaplan–Meier curves showing that high RARRES1 expression is a protective factor for overall survival in patients with luminal breast cancer in the METABRIC cohort. **(B)** RARRES1 expression is not associated with overall survival in patients with HER2+ breast cancer in the METABRIC Cohort. **(C)** RARRES 1 expression in patients with different subtype of breast cancer. **(D)** The correlation between RARRES1 expression and overall survival in 26 other cancer types based on TCGA database.

We further explored the role of *RARRES1* in pan-cancer. Kaplan–Meier survival analysis indicated that high *RARRES1* expression was also a protective factor for overall survival in patients with luminal breast cancer (Figure 6A). However, it was not related to overall survival in patients with HER2-positive breast cancer (Figure 6B). Notably, *RARRES1* expression level was significantly different between subtypes of breast cancer (Figure 6C). Specifically, *RARRES1* level was significantly higher in TNBC than in Luminal or HER2-positive breast cancer, and it was also significantly higher in HER2-positive breast cancer than in luminal breast cancer (Figure 6C). The correlation between *RARRES1* expression and overall survival was subsequently evaluated in 26 other cancer types based on the TCGA database. Univariate Cox regression suggested that high *RARRES1* level had a protective role in diffuse large B-cell lymphoma (DLBC, *p* = 0.006), mesothelioma (MESO, *p* = 0.005), skin cutaneous melanoma (SKCM, *p* = 0.002) and Sarcoma (SARC, *p* = 0.004), while it was associated with shorter overall survival in ovarian cancer (OV, *p* = 0.008), pancreatic adenocarcinoma (PAAD, *p* = 0.027), uterine corpus endometrial carcinoma (UCEC, *p* < 0.001), glioblastoma multiforme (GBM, *p* < 0.001), kidney renal clear cell carcinoma (KIRC, *p* < 0.001), and lower grade glioma (LGG, *p* < 0.001) (Figure 6D).

**FIGURE 7:**
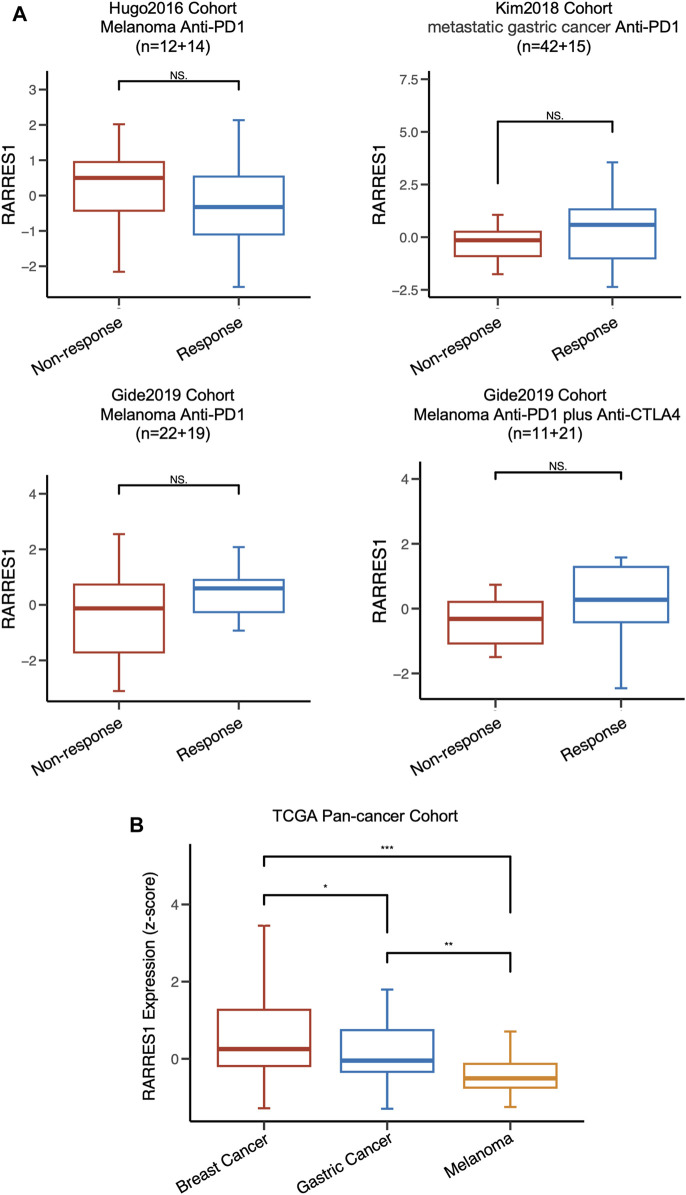
RARRES1 is not a predictor of immune response in melanoma and gastric cancer. **(A)** the distribution of RARRES1 expression level among different immunotherapy response patient cohorts. **(B)** RARRES1 expression among patients with breast cancer, gastric cancer, and melanoma in TCGA cohort.

We also investigated the predictive value of pre-treatment *RARRES1* level for the response to immunotherapy in four published cohorts with other types of cancer. Regrettably, *RARRES1* level was not associated with immunotherapy response in melanoma or gastric cancer (Figure 7A). This may be attributed to the lower expression levels of *RARRES1* in gastric cancer and melanoma (Figure 7B). In conclusion, *RARRES1* is a TNBC-specific predictor whose role is heterogeneous across cancer types.

## 4 Discussion

The tumor microenvironment consists of a variety of cellular and non-cellular components that play a critical role in the carcinogenesis and progression of cancer. [4] Characterizing the interactions between TME and cancer cells can help predict the efficacy of immunotherapy and develop novel anti-tumor treatments. [4] Single-cell sequencing techniques provide valuable tools for systematic profiling of cells in the TME (Huang and Zhang, 2021a; Mei et al., 2023; Zhang et al., 2023). Several studies have identified key cellular components or subpopulations associated with immune response in breast cancer. T cells and myeloid cells are the main infiltrating cell components of the tumor microenvironment in breast cancer (Wu et al., 2021). Pre-treatment CXCL13+ tumor-reactive T cells were associated with a favorable response to ICIs in TNBC, and were significantly expanded after treatment with chemotherapy combined with ICIs (Zhang et al., 2021; Liu et al., 2022). Overall macrophage infiltration is associated with unfavorable response to immunotherapy and poor survival, whereas there is heterogeneity in the role of distinct subpopulations of TAMs in the regulation of the immune response (Zhao et al., 2017). Conventional M1 and M2 markers were co-expressed in macrophage subsets in TNBC, and were not strongly associated with immune response and prognosis (Azizi et al., 2018; Zhang et al., 2021). In contrast, macrophage polarity as defined by *CXCL9*, *SPP1*, and *TREM2* had a remarkable correlation with the response to ICIs (Nalio Ramos et al., 2022; Bill et al., 2023). The ratio of *CXCL9* and *SPP1* was identified as a key determinant in regulating TME in multiple cancers (Bill et al., 2023). In responders to ICIs, lymphoid tissue inducer cells, follicular B cells, and cDC1s increased synergistically after treatment, which also suggests potentially roles for these cells in immune response (Zhang et al., 2021).

In the present study, we systematically profiled the cellular components of the TME in TNBC by scRNA-seq data, as well as identified signature genes of pro-inflammatory immune cells, including CXCL13+ tumor-reactive T cells, activated T cells, effector T cells, CXCL9+ TAMs, NK cells, and cDC1. We also established a method for quantitatively evaluation of the tumor immune microenvironment, TME-hot score, which can be used not only for TNBC but also for other types of cancer in the future. The TME-hot score allows us to apply the characteristics of each cell type depicted by scRNA-seq to bulk RNA-seq data, which comes with advantages of low cost and large sample size. Furthermore, by screening cancer cell-intrinsic genes associated with TME-hot score, we identified *RSRRES1* as a predictor of prognosis and immune response in TNBC. *RARRES1* could also be a potential target for immunotherapy, which requires further studies on the mechanism of its interaction with TME in the future.

*RARRES1*, Retinoic acid receptor responder 1, was thought to be a membrane protein which regulates metabolism, differentiation, and apoptosis of cell lines *in vitro* (Oldridge et al., 2013; Maimouni et al., 2019). Although *RARRES1* has been identified as a tumor suppressor gene in several studies, however, the relationship between *RARRES1* and prognosis is controversial. In prostate cancer, epigenetic silencing of *RARRES1* was shown to be associated with poor prognosis (Kloth et al., 2012; Oldridge et al., 2013). However, several studies suggested that high *RARRES1* expression is significantly correlated with poorer clinical outcomes in patients with clear cell renal cell carcinoma and inflammatory breast cancers (Wang et al., 2013; Geng et al., 2022). The present study demonstrated that *RARRES1* level is positively related with favorable prognosis and “hot” TME in two large TNBC cohorts. A recent study revealed that *RARRES1* exerts an anti-tumor effect by promoting *ICAM1* expression and inducing M1 macrophage activation in renal cancer *in vitro* (Geng et al., 2022). Therefore, the role of *RARRES1* and its effect on TME needs to be further confirmed by *in vivo* experiments.

There are several limitations in our study that should be noted. Although the role of *RARRES1* in prognosis and response to immunotherapy was validated in two published cohorts, further illumination through experimental and clinical studies remains necessary. Comprehensive functional experiments would contribute to understanding the detailed role of *RARRES1* in the interactions between tumor cells and TME. Meanwhile, the response to ICIs of patients in the BioKey cohort was determined by T cell expansion, so the predictive role of *RARRES1* on immunotherapy needs to be further confirmed in prospective clinical trials. Furthermore, the characterization of TME in TNBC may be limited by the small sample size of the scRNA-seq cohort.

In conclusion, we comprehensively resolved cell types and subpopulations in the TME of TNBC by scRNA-seq. We also proposed a method for quantitatively assessment of the TME immune profile and provided a framework for identifying factors associated with TME through integrated analysis. In addition, we characterized high *RSRRES1* expression as a predictor of favorable prognosis and response to ICIs in TNBC. The role of *RARRES1* requires to be further clarified in the future by functional experiments and clinical trials.

## Data Availability

The original contributions presented in the study are included in the article/Supplementary Material, further inquiries can be directed to the corresponding authors.

## Abbreviations

DEGs, differentially expressed genes; ER, estrogen receptor; ES, Enrichment score; GEO, Gene Expression Omnibus; HER2, human epidermal growth factor receptor 2; ICIs, immune checkpoint inhibitors; METABRIC, Molecular Taxonomy of Breast Cancer International Consortium; PCA, principal component analysis; PR, progesterone receptor; scRNA-seq, single-cell RNA sequencing; ssGSEA, single-sample gene set enrichment analysis; TCGA, the Cancer Genome Atlas; TNBC, triple-negative breast cancer; TAMs, tumor-associated macrophages; TME, tumor microenvironment; UMAP, uniform manifold approximation and projection.
