# Supplementary figures and images for: Integrative analysis identifies cancer cell-intrinsic RARRES1 as a predictor of prognosis and immune response in triple-negative breast cancer
Source: Front Genet. 2024 Mar 12;15:1360507. doi: 10.3389/fgene.2024.1360507 (PMC10963550; doi:10.3389/fgene.2024.1360507)

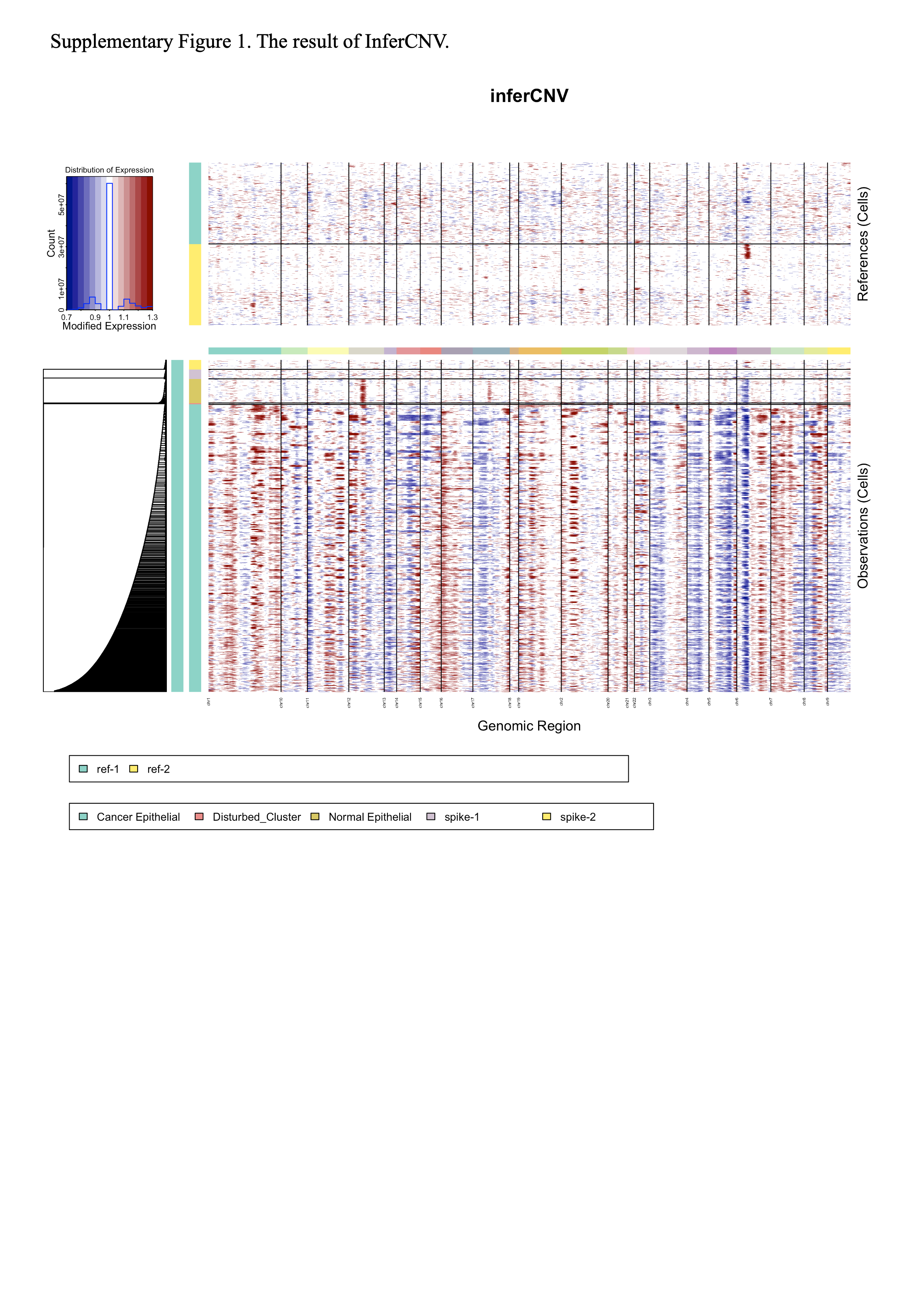

Supplement: Supplementary file 1 [file Image1.TIFF]

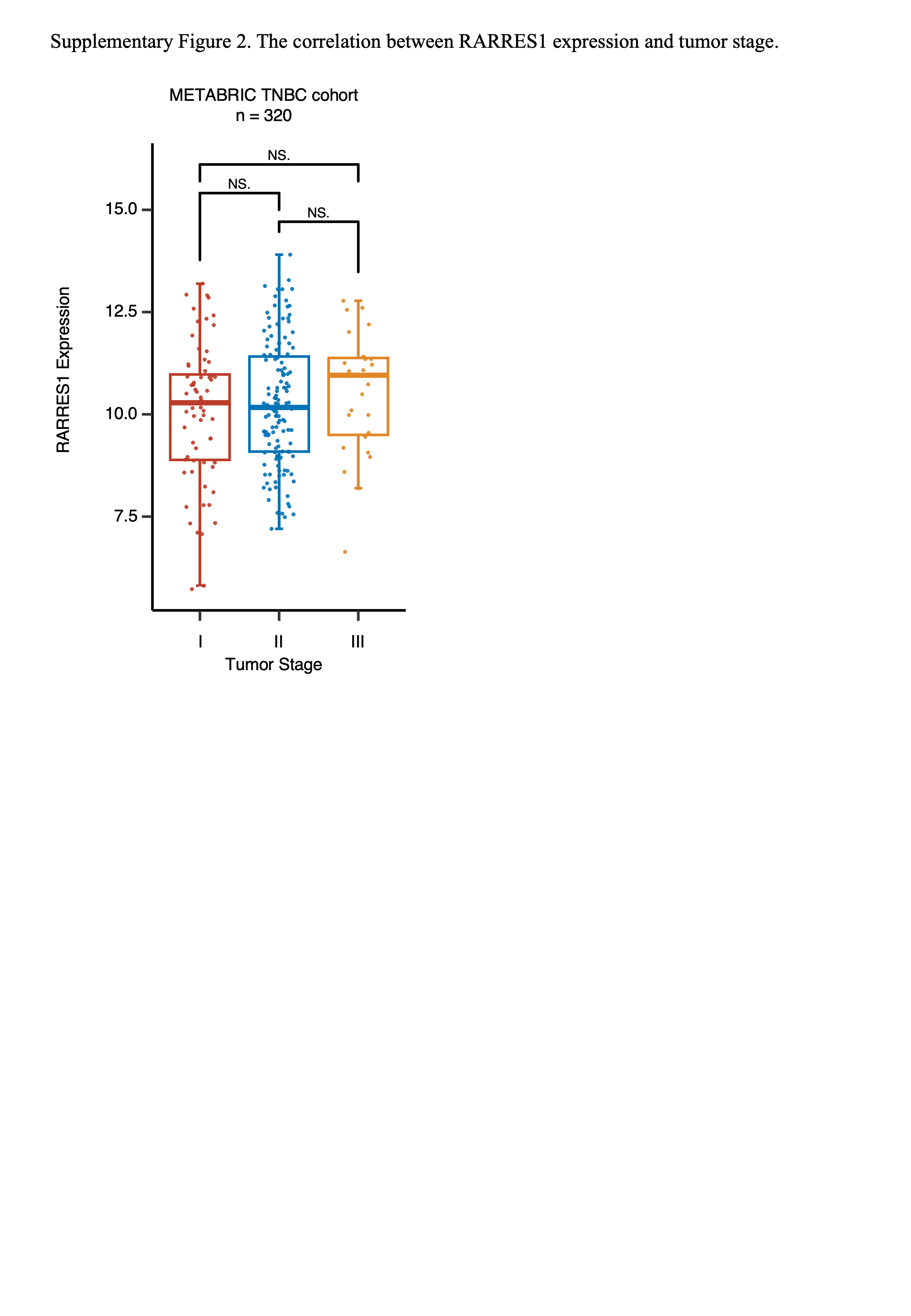

Supplement: Supplementary file 3 [file Image2.TIFF]
